# Synthesis and structure evaluation of new complex butylarylpiperazin-1-yl derivatives

**DOI:** 10.1007/s00044-013-0740-z

**Published:** 2013-09-03

**Authors:** Daniel Szulczyk, Anna Bielenica, Michał A. Dobrowolski, Łukasz Dobrzycki, Mariola Krawiecka, Bożena Kuran, Marta Struga

**Affiliations:** 1 Department of Medical Chemistry, Faculty of Medicine, Medical University of Warsaw, Oczki 3 Street, 02-007 Warsaw, Poland; 2 Faculty of Chemistry, University of Warsaw, Pasteura 1 Street, 02-093 Warsaw, Poland

**Keywords:** 5-HT_1A_ receptor, Arylpiperazines, X-ray crystallography, Diels–Alder reaction

## Abstract

A series of arylpiperazine derivatives of 1,16-diphenyl-19-azahexacyclo-[14.5.1.0^2,15^.0^3,8^.0^9,14^.0^17,21^]docosa-2,3,5,7,8,9,11,13,14-nonaene-18,20,22-trione and 4,10-diphenyl-1*H*,2*H*,3*H*,5*H*-indeno[1,2-*f*]isoindole-1,3,5-trione was synthesized. The pharmacological profile of compound **4** at the 5-HT_1A_ receptor was measured by binding assay. The title compounds were tested in cell-based assay against the human immunodeficiency virus type-1. The X-ray crystallographic studies of derivatives **2**, **6**, **7**, **11**, **19**, and **20** were presented.

## Introduction

**Fig. 1:**
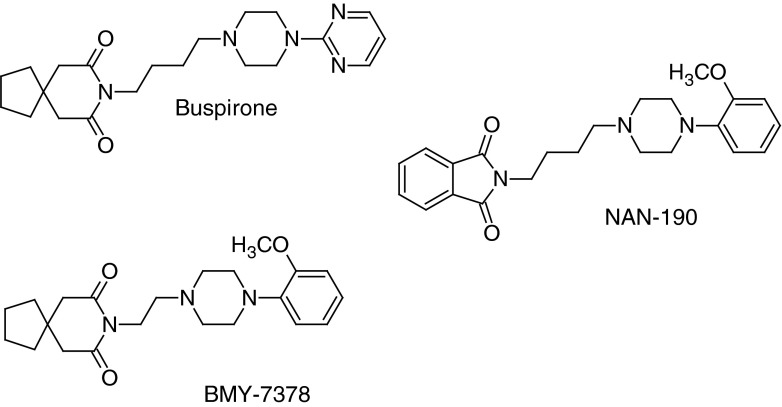
Some representative 5-HT_1A_ receptor ligands

The literature survey shows that many ligands of serotonin 5-HT_1A_, 5-HT_2A_, and 5-HT_7_ receptors contain a flexible hydrocarbon chain of different lengths, attached to an arylpiperazine moiety that is the pharmacophore group (Fig. 1) (Lewgowd *et al*., 2011; Czopek *et al*., 2010; Bojarski, 2006; Leopoldo, 2004). The pharmacophore group is recognized not only by metabotropic serotonin receptor binding sites, but also by those of D_2_-dopaminergic (González-Gómez *et al*., 2003) and α_1_-adrenergic receptors (Prandi *et al*., 2012).

Using quantitative structure–activity relationship analysis, the “rule of five” scheme was worked out for orally administrated drugs (Lipinski *et al*., 1997; Kerns and Di, 2008). According to authors, the drugs that cross the blood–brain barrier are those of molecular mass lower than 450 u and of theoretical partition coefficient *n*-octanol/water (log*P*) being in the range of 1–4 or log*D*_7.4_ 1–3. The biological barrier permeability is also determined by the following important parameters: numbers of hydrogen bond donors and acceptors in the potential medicine’s structure (HBD maximum 4 and HBA less than 6), polar surface area (PSA) correlated with them [expected value is less than 60–70 Å^2^ (Oprea, 2002)], as well as compound’s solubility (log*S* greater than 60 μg/cm^3^). Proper drug permeability makes it possible to cross the barrier and to reach the regions of a drug’s action.

In last two decades, a number of binding modes of long-chain arylpiperazine derivatives to 5-HT_1A_ (Lewgowd *et al*., 2011; Nowak *et al*., 2006), 5-HT_2A_ (Klabunde and Evers, 2005; Bronowska *et al*., 2001), and 5-HT_7_ (Kim *et al*., 2012; López-Rodríguez *et al*., 2003) receptors have been proposed. The ionic interaction between the protonated nitrogen of the piperazine ring of a ligand and Asp3.32 residue of the receptor (Nowak *et al*., 2006; Vermeulen *et al*., 2003; Roth *et al*., 1997) constituted a main essential interaction. The hydrophobic terminal imide or amide group, the hydrocarbon linker, and an aromatic ring bound to the piperazine moiety are placed in a hydrophobic pocket composed of aromatic and/or aliphatic amino acids side chains (Kim *et al*., 2012; Varin *et al*., 2010; Lepailleur *et al*., 2005). The flexible chain of *N*-(4-arylpiperazin-1-yl-alkyl)substituted derivatives can adopt one of the two main conformations: extended or bent. The results of geometry optimization (Lewgowd *et al*., 2011) proved that conformers with extended spacer are preferred in a solution, whereas in vacuum bent geometries predominate. Theoretical calculations determine minimum energy for extended linker conformations also in solid state and for complexes with a receptor (Siracusa *et al*., 2008). According to pharmacophore model of the 5-HT_1A_ receptor (Chilmonczyk *et al*., 1997; Bronowska *et al*., 2001), a folded conformer promotes high affinity for the 5-HT_1A_ receptor. It is known that ligand binding can lead to a change in the conformation of the receptor protein, however, also in the ligand itself (Sylte *et al*., 2001). In addition, the role of the solvent molecules is quite difficult to explain—they can take part in a ligand—receptor H-bond formation, be involved in the process of a receptor activation or influence entropy effects (Pardo *et al*., 2007).

This paper reports synthesis and biological activity of compounds purposely designed to combine the bulky hydrophobic imide ring with alkyl linker bearing different substituents. The collected group of arylpiperazine derivatives can be used for further investigations concerning ligand-5-HT receptor interactions. For this reason X-ray crystallographic studies of derivatives **2**, **6**, **7**, **11**, **19**, and **20** were described. The molecular descriptors for selected arylpiperazine derivatives were presented. The pharmacological profile of the compound **4** was evaluated for its affinity to the 5-HT_1A_ receptor. It was reported, that cytotoxicity of aromatic, high-volume arylpiperazine derivatives is low (Filosa *et al*., 2007; Ananda Kumar *et al*., 2009), and they act as anti-HIV-1 agents (Yang *et al*., 2010), cytotoxicity and anti-HIV activity of selected derivatives were examined.

## Materials and methods

### Chemistry

All chemicals and solvents were purchased from Aldrich. Melting points were determined on an Electrothermal Digital Melting Point Apparatus and are uncorrected. The NMR spectra were recorded on a Bruker AVANCE DMX400 spectrometer, operating at 300 MHz (^1^H NMR) and 75 MHz (^13^C NMR). The chemical shift values are expressed in ppm relative to TMS as an internal standard. Mass spectral electrospray ionization (ESI) measurements were carried out on a Mariner Perspective—Biosystem instrument with TOF detector. The spectra were obtained in the positive ion mode with a declustering potential 140–300 V. Elemental analyses were recorded on a CHN model 2400 Perkin-Elmer. TLC was carried out using silica gel 60 F_254_ with layer thickness of 0.25 mm (Merck) and the results were visualized using UV lamp at 254 nm. Column chromatography was carried out using silica gel 60 (200–400 mesh, Merck) and chloroform/methanol (19.5:0.5 vol) mixture as eluent.

### 1,16-Diphenyl-19-azahexacyclo[14.5.1.0^2,15^.0^3,8^.0^9,14^.0^17,21^]docosa-2,3,5,7,8,9,11,13,14-nonaene-18,20,22-trione (**1**)

The mixture of 2.14 g (0.004 mol) of 1,3-diphenyl-2*H*-cyclopenta[*l*]phenanthren-2-one (“Phencyclone”) was suspended in 75 ml of benzene and 0.48 g (0.005 mol) of maleimide was added. After refluxing time of 8 h the residue was evaporated, and the residue was purified by column chromatography (chloroform:methanol 9.5:0.5 vol). The combined fractions were condensed to dryness to give 1.86 g (87 %) of **(1)**, m.p. 327–328 °C. ^1^H NMR (DMSO-*d*_6_) *δ* (ppm): 11.04 (s, 1H, NH), 8.85 (d, 2H, CH_arom._, *J* = 8.4 Hz), 8.24 (d, 2H, CH_arom._, *J* = 7.8 Hz), 7.73 (t, 2H, CH_arom._, *J* = 7.2 Hz), 7.59–7.51 (m, 4H, CH_arom._), 7.41 (t, 2H, CH_arom._, *J* = 7.5 Hz), 7.22 (t, 2H, CH_arom._, *J* = 7.8 Hz), 7.14 (d, 2H, CH_arom._, *J* = 7.8 Hz), 7.04 (d, 2H, CH_arom._, *J* = 5.7 Hz), 4.65 (s, 2H, CH). ^13^C NMR (DMSO-*d*_6_) *δ* (ppm): 197.12, 173.09, 173.05, 134.03, 133.98, 133.48, 133.22, 133.76 (2C), 132.37, 132.12, 132.09, 132.06, 132.00, 131.83, 131.62, 131.47, 130.49, 130.21, 129.75, 129.68 (2C), 128.63, 128.54, 127.96, 126.84, 126.78, 122.35, 122.31, 63.65, 63.59, 45.25, 45.20. Anal. Calcd. for C_33_H_21_NO_3_: C, 82.45; H, 4.38; N, 2.92. Found: C, 82.40; H, 3.00; N, 4.40.

### 19-(4-Bromobutyl)-1,16-diphenyl-19-azahexacyclo-[14.5.1.0^2,15^.0^3,8^.0^9,14^.0^17,21^]-docosa-2,3,5,7,8,9,11,13,14-nonaene-18,20,22-trione (**2**)

A mixture of imide **(1)** (1.41 g, 0.003 mol), 1,4-dibromobutane (0.7 ml, 0.006 mol), anhydrous K_2_CO_3_ (1.39 g), and catalytic amount of KI were refluxed in acetonitrile for 24 h. Then the solvent was removed on a rotary evaporator and the oily residue was purified by column chromatography (chloroform:methanol 9.5:0.5 vol). The combined fractions were condensed to dryness to give 1.36 g (86 %) of **(2)**, m.p. 286–289 °C. ^1^H NMR (DMSO-*d*_6_) *δ* (ppm): 8.84 (d, 2H, CH_arom._, *J* = 9.0 Hz), 8.27 (d, 2H, CH_arom._, *J* = 8.4 Hz), 7.75 (t, 2H, CH_arom._, *J* = 8.1 Hz), 7.59–7.52 (m, 4H, CH_arom._), 7.43 (t, 2H, CH_arom._, *J* = 8.7 Hz), 7.25–7.14 (m, 4H, CH_arom._), 7.01 (d, 2H, CH_arom._, *J* = 7.5 Hz), 4.61 (s, 2H, CH), 2.87–2.78 (m, 2H, CH_2_), 2.11–2.07 (m, 2H, CH_2_), 1.24–1.21 (m, 2H, CH_2_), 0.49–0.43 (m, 2H, CH_2_). ^13^C NMR (DMSO-*d*_6_) *δ* (ppm): 197.09, 173.12, 173.01, 134.11, 133.88, 133.51 (2C), 133.28, 133.39, 132.32, 132.17, 132.04, 132.00, 131.90, 131.87, 131.65, 131.36, 130.27, 130.19, 129.83, 129.69, 129.66, 128.52, 128.47, 127.89, 126.72, 126.68, 122.33, 122.30, 63.68, 63.61, 45.31, 45.28, 44.89, 32.79, 28.74, 28.53. ESI MS: *m/z* = 638.0 [M+H]^+^ (100 %).

### General method for the preparation of arylpiperazine derivatives of 19-(4-bromobutyl)-1,16-diphenyl-19-azahexacyclo[14.5.1.0^2,15^.0^3,8^.0^9,14^.0^17,21^]docosa-2,3,5,7,8,9,11,13,14-nonaene-18,20,22-trione (**3**–**9**)

A mixture of derivative **(2)** (0.3 g, 0.002 mol) and the corresponding amine (0.004 mol), anhydrous K_2_CO_3_ (0.3 g), and catalytic amount of KI were refluxed in acetonitrile for 30 h. Then the mixture was filtered off and the solvent was evaporated. The gray residue was purified by column chromatography (chloroform:methanol 9.5:0.5 vol) and/or crystallized from methanol. Obtained compounds were converted into their hydrochlorides. The solid product was dissolved in methanol saturated with gaseous HCl. The hydrochloride was precipitated by addition of diethyl ether. The crude product was crystallized from an appropriate solvent.

### 1,16-Diphenyl-19-(4-(4-pyridin-2-ylpiperazin-1-yl)butyl)-19-azahexacyclo-[14.5.1.0^2,15^.0^3,8^.0^9,14^.0^17,21^]docosa-2,3,5,7,8,9,11,13,14-nonaene-18,20,22-trione (**3**)

Yield: 67 %, m.p. 200–203 °C. ^1^H NMR (DMSO-*d*_6_) *δ* (ppm): 8.81 (d, 2H, CH_arom._, *J* = 8.7 Hz), 8.27 (d, 2H, CH_arom._, *J* = 8.1 Hz), 8.09–8.06 (m, 1H, CH_arom._), 7.74 (t, 2H, CH_arom._, *J* = 7.8 Hz), 7.57–7.40 (m, 7H, CH_arom._), 7.36–7.14 (m, 4H, CH_arom._), 7.05 (d, 2H, CH_arom._, *J* = 9.3 Hz), 6.75 (d, 1H, CH_arom._, *J* = 8.7 Hz), 6.60 (d–d, 1H, CH_arom._, *J*_1_ = 5.1 Hz, *J*_2_ = 5.4 Hz), 4.67 (s, 2H, CH), 3.78 (s, 1H, CH_2_), 3.31–2.72 (m, 3H, CH_2_), 3.05 (s, 1H, CH_2_), 2.92 (s, 1H, CH_2_), 2.05 (t, 4H, CH_2_, *J* = 2.1 Hz), 1.44 (t, 2H, CH_2_, *J* = 7.2 Hz), 1.24–1.22 (m, 1H, CH_2_), 0.88–0.83 (m, 1H, CH_2_), 0.33–0.23 (m, 2H, CH_2_). ^13^C NMR (DMSO-*d*_6_) *δ* (ppm): 197.17, 173.08, 173.02, 157.48, 147.68, 137.35, 134.24, 133.73, 133.68, 133.35, 133.30, 132.12 (3C), 132.07, 132.02, 132.00, 131.87, 131.69, 131.51, 130.31, 130.12, 129.99, 129.84, 129.73, 128.47, 128.32, 127.77, 126.58, 126.49, 122.41, 122.19, 119.83, 108.92, 63.75, 63.72, 50.87, 50.43, 48.58, 48.49, 45.34, 45.32, 44.86, 32.69, 28.81, 28.73. ESI MS: *m/z* = 697.1 [M+H]^+^ (100 %).

### 19-(4-(4-(2-(Methyloxy)phenyl)piperazin-1-yl)butyl)-1,16-diphenyl-19-azahexa-cyclo[14.5.1.0^2,15^.0^3,8^.0^9,14^.0^17,21^]docosa-2,3,5,7,8,9,11,13,14-nonaene-18,20,22-trione (**4**)

Yield: 71 %, m.p. 197–200 °C. ^1^H NMR (DMSO-*d*_6_) *δ* (ppm): 8.83 (d, 2H, CH_arom._, *J* = 8.4 Hz), 8.27 (d, 2H, CH_arom._, *J* = 7.8 Hz), 7.74 (t, 2H, CH_arom._, *J* = 7.8 Hz), 7.58–7.52 (m, 4H, CH_arom._), 7.42 (t, 2H, CH_arom._, *J* = 7.5 Hz), 7.24–7.14 (m, 4H, CH_arom._), 7.10 (d, 2H, CH_arom._, *J* = 8.7 Hz), 6.92–6.83 (m, 4H, CH_arom._), 4.68 (s, 2H, CH), 3.75 (s, 3H, OCH_3_), 2.78–2.72 (m, 7H, CH_2_), 2.17–2.12 (m, 4H, CH_2_), 1.44 (t, 3H, CH_2_, *J* = 7.2 Hz), 1.23–1.16 (m, 1H, CH_2_), 1.05 (t, 1H, CH_2_, *J* = 6.9 Hz). ^13^C NMR (DMSO-d_6_) *δ* (ppm): 197.14, 173.11, 173.09, 157.44, 147.52, 142.74, 137.31, 134.27, 133.79, 133.66, 133.31 (2C), 133.30, 132.16 (2C), 132.03, 132.01, 131.96, 131.83, 131.68, 131.57, 130.34, 130.05, 129.94, 129.81, 129.78, 128.44, 128.29, 127.68, 126.53, 126.47, 122.46, 122.21, 119.80, 108.87, 63.74, 63.71, 55.12, 50.85, 50.46, 48.53, 48.47, 45.35, 45.31, 44.88, 32.67, 28.78, 28.74. ESI MS: *m/z* = 726.1 [M+H]^+^ (100 %).

### 1,16-Diphenyl-19-(4-(4-phenylpiperazin-1-yl)butyl)-19-azahexacyclo-[14.5.1.0^2,15^.0^3,8^.0^9,14^.0^17,21^]docosa-2,3,5,7,8,9,11,13,14-nonaene-18,20,22-trione (**5**)

Yield: 69 %, m.p. 202–203 °C. ^1^H NMR (DMSO-*d*_6_) *δ* (ppm): 8.71 (d, 2H, CH_arom._, *J* = 8.1 Hz), 8.31 (d, 2H, CH_arom._, *J* = 8.1 Hz), 7.62–7.69 (m, 2H, CH_arom._), 7.64–7.48 (m, 7H, CH_arom._), 7.45–7.37 (m, 3H, CH_arom._), 7.22–7.14 (m, 6H, CH_arom._), 7.08–7.04 (m, 1H, CH_arom._), 4.48 (s, 2H, CH), 3.51–3.42 (m, 4H, CH_2_), 3.27–3.23 (m, 3H, CH_2_), 3.13–2.95 (m, 4H, CH_2_), 2.63–2.61 (m, 2H, CH_2_), 2.35–2.29 (m, 3H, CH_2_). ^13^C NMR (DMSO-*d*_6_) *δ* (ppm): 197.23, 173.17, 173.09, 157.53, 147.75, 137.42, 134.33, 133.82, 133.79, 133.41, 133.32, 132.17, 132.11, 132.06, 132.03, 131.92, 131.77 (2C), 131.58, 130.43, 130.18, 129.98, 129.89, 129.78 (2C), 128.51, 128.39, 127.81, 126.62, 126.53, 122.48, 122.22, 119.86, 115.37, 115.29, 63.81, 63.78, 50.90, 50.62, 48.64, 48.54, 45.48, 45.46, 44.93, 32.70, 28.84, 28.77. ESI MS: *m*/*z* = 696.2 [M+H]^+^ (100 %).

### 19-(4-(4-(4-Chlorophenyl)piperazin-1-yl)butyl)-1,16-diphenyl-19-azahexacyclo-[14.5.1.0^2,15^.0^3,8^.0^9,14^.0^17,21^]docosa-2,3,5,7,8,9,11,13,14-nonaene-18,20,22-trione (**6**)

Yield: 92 %, m.p. 220–221 °C. ^1^H NMR (DMSO-*d*_6_) *δ* (ppm): 8.15 (d, 2H, CH_arom._, *J* = 8.4 Hz), 8.27 (d, 2H, CH_arom._, *J* = 7.5 Hz), 7.74 (t, 2H, CH_arom._, *J* = 7.8 Hz), 7.57–7.52 (m, 4H, CH_arom._), 7.42 (t, 2H, CH_arom._, *J* = 7.5 Hz), 7.24–7.13 (m, 6H, CH_arom._), 7.02 (d, 2H, CH_arom._, *J* = 8.7 Hz), 6.88 (d, 2H, CH_arom._, *J* = 9.3 Hz), 4.67 (s, 2H, CH), 3.49–3.43 (m, 4H, CH_2_), 3.28–3.20 (m, 3H, CH_2_), 3.15–2.99 (m, 4H, CH_2_), 2.69–2.59 (m, 2H, CH_2_), 2.37–2.30 (m, 3H, CH_2_). ^13^C NMR (DMSO-*d*_6_) *δ* (ppm): 197.21, 173.11, 173.06, 157.50, 147.74, 137.41, 134.36, 133.81, 133.78, 133.43, 133.33, 132.15, 132.12, 132.07, 132.04, 131.95, 131.72, 131.68, 131.56, 130.46, 130.12, 129.97, 129.84, 129.73 (2C), 128.59, 128.37, 127.85, 126.65, 126.54, 122.47, 122.25, 119.83, 115.39, 115.28, 63.80, 63.76, 50.91, 50.67, 48.68, 48.57, 45.42, 45.40, 44.96, 32.75, 28.86, 28.73. ESI MS: *m*/*z* = 730.1 [M+H]^+^ (100 %).

### 19-(4-(4-(2-Fluorophenyl)piperazin-1-yl)butyl)-1,16-diphenyl-19-azahexacyclo-[14.5.1.0^2,15^.0^3,8^.0^9,14^.0^17,21^]docosa-2,3,5,7,8,9,11,13,14-nonaene-18,20,22-trione (**7**)

Yield: 87 %, m.p. 205–207 °C. ^1^H NMR (DMSO-*d*_6_) *δ* (ppm): 8.83 (d, 2H, CH_arom._, *J* = 8.4 Hz), 8.28 (d, 2H, CH_arom._, *J* = 7.2 Hz), 7.74 (t, 2H, CH_arom._, *J* = 7.2 Hz), 7.58–7.52 (m, 4H, CH_arom._), 7.42 (t, 2H, CH_arom._, *J* = 7.8 Hz), 7.24–7.14 (m, 4H, CH_arom._), 7.10–6.95 (m, 6H, CH_arom._), 4.68 (s, 2H, CH), 3.39–3.36 (m, 2H, CH_2_), 3.11–3.07 (m, 2H, CH_2_), 3.03–2.93 (m, 4H, CH_2_), 2.73–2.71 (m, 4H, CH_2_), 2.14–2.10 (m, 4H, CH_2_). ^13^C NMR (DMSO-*d*_6_) *δ* (ppm): 197.20, 173.41, 173.35, 157.56, 147.54, 137.61, 134.41, 133.87, 133.79, 133.54, 133.49, 132.28, 132.17, 132.08, 132.02, 131.90, 131.76, 131.61, 131.55, 130.40, 130.17, 129.93, 129.82, 129.73, 129.70, 128.53, 128.34, 127.82, 126.69, 126.51, 122.48, 122.23, 119.88, 115.33, 115.27, 63.81, 63.74, 50.98, 50.63, 48.62, 48.54, 45.43, 45.41, 44.96, 32.72, 28.82, 28.79. ESI MS: *m*/*z* = 714.2 [M+H]^+^ (100 %).

### 19-(4-(4-(4-Acetylphenyl)piperazin-1-yl)butyl)-1,16-diphenyl-19-azahexacyclo-[14.5.1.0^2,15^.0^3,8^.0^9,14^.0^17,21^]docosa-2,3,5,7,8,9,11,13,14-nonaene-18,20,22-trione (**8**)

Yield: 77 %, m.p. 202–204 °C. ^1^H NMR (DMSO-*d*_6_) *δ* (ppm): 8.82 (d, 2H, CH_arom._, *J* = 8.1 Hz), 8.28 (d, 2H, CH_arom._, *J* = 7.8 Hz), 7.80–7.72 (m, 4H, CH_arom._), 7.54 (t, 2H, CH_arom._, *J* = 7.2 Hz), 7.42 (t, 2H, CH_arom._, *J* = 7.5 Hz), 7.22 (t, 2H, CH_arom._, *J* = 7.8 Hz), 7.15 (d, 2H, CH_arom._, *J* = 7.8 Hz), 7.03 (d, 2H, CH_arom._, *J* = 8.1 Hz), 6.92 (d, 2H, CH_arom._, *J* = 9.3 Hz), 4.68 (s, 2H, CH), 3.52–3.44 (m, 4H, CH_2_), 3.16 (t, 4H, CH_2_, *J* = 4.2 Hz), 2.77 (t, 2H, CH_2_, *J* = 6.9 Hz), 2.44 (s, 3H, COCH_3_), 2.10–2.07 (m, 4H, CH_2_), 1.46 (t, 2H, CH_2_, *J* = 6.9 Hz). ^13^C NMR (DMSO-*d*_6_) *δ* (ppm): 197.23, 186.59, 173.39, 173.37, 157.51, 147.59, 137.62, 134.57, 133.89, 133.85, 133.69, 133.57, 132.55, 132.34, 132.17, 132.11, 131.92, 131.85, 131.69, 131.57, 130.46, 130.38, 129.90, 129.83, 129.77, 129.72, 128.59, 128.30, 127.75, 126.61, 126.54, 122.47, 122.26, 119.82, 115.36, 115.34, 63.85, 63.82, 50.78, 50.68, 48.61, 48.59, 45.47, 45.44, 44.92, 32.77, 28.90, 28.83, 26.82. ESI MS: *m*/*z* = 738.6 [M+H]^+^ (100 %).

### 1,16-Diphenyl-19-(4-(4-(2-(trifluoromethyl)phenyl)piperazin-1-yl)butyl)-19-azahexa-cyclo[14.5.1.0^2,15^.0^3,8^.0^9,14^.0^17,21^]docosa-2,3,5,7,8,9,11,13,14-nonaene-18,20,22-trione (**9**)

Yield: 84 %, m.p. 211–212 °C. ^1^H NMR (DMSO-*d*_6_) *δ* (ppm): 8.78 (d, 2H, CH_arom._, *J* = 8.4 Hz), 8.30 (d, 2H, CH_arom._, *J* = 7.8 Hz), 7.74 (t, 2H, CH_arom._, *J* = 6.3 Hz), 7.69–7.60 (m, 3H, CH_arom._), 7.54 (t, 3H, CH_arom._, *J* = 6.3 Hz), 7.48–7.40 (m, 4H, CH_arom._), 7.18–7.14 (m, 2H, CH_arom._), 4.48 (s, 2H, CH), 3.95–3.91 (m, 3H, CH_2_), 3.61–3.37 (m, 10H, CH_2_), 3.22–3.17 (m, 3H, CH_2_), 3.01–2.92 (m, 4H, CH_2_). ^13^C NMR (DMSO-*d*_6_) *δ* (ppm): 197.19, 173.12, 173.05, 157.51, 147.74, 137.40, 134.36, 133.88, 133.77, 133.43, 133.37, 132.15, 132.10, 132.04, 132.01, 131.99, 131.78 (2C), 131.54, 130.48, 130.13, 129.92, 129.86, 129.71 (2C), 128.53, 128.37, 127.86, 126.66, 126.51, 123.92, 122.45, 122.18, 119.83, 115.34, 115.28, 63.80, 63.78, 61.17, 50.92, 50.68, 48.62, 48.59, 45.44, 45.41, 44.97, 32.76, 31.28, 28.87, 28.73. ESI MS: *m*/*z* = 792.2 [M+H]^+^ (100 %).

### 10-Diphenyl-1*H*,2*H*,3*H*,5*H*-indeno[1,2-*f*]isoindole-1,3,5-trione (**10**)

The mixture of 2.06 g (0.006 mol) of 1,3-diphenylcyclopenta[*a*]indene-2,8-dione (“Indanocyclone”) was suspended in 75 ml of benzene and 0.65 g (0.006 mol) of maleimide was added. After refluxing time of 16 h the yellow residue was evaporated. Next it was purified by column chromatography (chloroform:methanol 9.5:0.5 vol). The combined fractions were condensed to dryness to give 1.50 g (73 %) of **(10)**, m.p. 223–225 °C. ^1^H NMR (CDCl_3_) *δ* (ppm): 7.60 (d, 2H, CH_arom._, *J* = 2.7 Hz), 7.59–7.58 (m, 2H, CH_arom._), 7.52 (d, 2H, CH_arom._, *J* = 2.1 Hz), 7.51–7.49 (m, 2H, CH_arom._), 7.45 (d, 2H, CH_arom._, *J* = 2.1 Hz), 7.44–7.40 (m, 4H, CH_arom._). ^13^C NMR (CDCl_3_) *δ* (ppm): 190.91, 165.89, 165.73, 149.69, 141.97, 139.37, 135.58, 135.52, 135.14, 134.81, 134.24, 131.59, 130.57, 130.54, 129.87, 129.34, 129.28 (2C), 129.09 (3C), 128.59 (2C), 127.91 (2C), 124.59, 124.54. ESI MS: *m*/*z* = 424.1 [M+Na]^+^ (100 %).

### 2-(4-Bromobutyl)-4,10-diphenyl-1*H*,2*H*,3*H*,5*H*-indeno[1,2-*f*]isoindole-1,3,5-trione (**11**)

A mixture of imide **(10)** (2.64 g, 0.006 mol), 1,4-dibromobutane (1.5 ml, 0.012 mol), anhydrous K_2_CO_3_ (2.51 g), and catalytic amount of KI were refluxed in acetonitrile for 14 h. Then the solvent was removed on a rotary evaporator and the dark yellow solid residue was purified by column chromatography (chloroform:methanol 9.5:0.5 vol). The combined fractions were condensed to dryness to give 2.44 g (92 %) of **(11)**, m.p. 241–242 °C. ^1^H NMR (CDCl_3_) *δ* (ppm): 7.63–7.59 (m, 3H, CH_arom._), 7.56–7.55 (m, 2H, CH_arom._), 7.53–7.50 (m, 4H, CH_arom._), 7.48–7.41 (m, 5H, CH_arom._), 3.72 (q, 2H, CH_2_, *J* = 7.2 Hz), 3.54 (t, 2H, CH_2_, *J* = 6.9 Hz), 3.35 (t, 2H, CH_2_, *J* = 6.3 Hz), 1.26–1.21 (m, 2H, CH_2_). ^13^C NMR (CDCl_3_) *δ* (ppm): 190.21, 165.67, 165.49, 148.11, 141.34, 137.49, 135.09, 134.80, 134.26, 134.04, 133.87, 132.08, 130.52, 129.75, 129.37 (3C), 128.79 (3C), 128.51 (2C), 128.17, 127.14 (2C), 124.03, 123.48, 36.63, 34.50, 29.57, 26.48. ESI MS: *m*/*z* = 560.1 [M+Na]^+^ (100 %).

### General method for the preparation of arylpiperazine derivatives of 2-(4-bromobutyl)-4,10-diphenyl-1*H*,2*H*,3*H*,5*H*-indeno[1,2-*f*]isoindole-1,3,5-trione (**12**–**19**)

A mixture of derivative **(11)** (0.3 g, 0.0005 mol) and the corresponding amine (0.001 mol), anhydrous K_2_CO_3_ (0.3 g), and catalytic amount of KI were refluxed in acetonitrile for 30 h. Then the mixture was filtered off and the solvent was evaporated. The yellow residue was purified by column chromatography (chloroform:methanol 9.5:0.5 vol) and/or crystallized from methanol. Obtained compounds were converted into their hydrochlorides. The solid product was dissolved in methanol saturated with gaseous HCl. The hydrochloride was precipitated by addition of diethyl ether. The crude product was crystallized from appropriate solvent.

### 4,10-Diphenyl-2-[4-(4-phenylpiperazin-1-yl)butyl]-1*H*,2*H*,3*H*,5*H*-indeno[1,2-*f*]isoindole-1,3,5-trione (**12**)

Yield: 87 %, m.p. 231–232 °C. ^1^H NMR (DMSO-*d*_6_) *δ* (ppm): 7.61 (t, 3H, CH_arom._, *J* = 3.6 Hz), 7.56–7.44 (m, 8H, CH_arom._), 7.40–7.31 (m, 2H, CH_arom._), 7.28–7.23 (m, 2H, CH_arom._), 6.98 (d, 2H, CH_arom._, *J* = 8.1 Hz), 6.86 (t, 1H, CH_arom._, *J* = 7.2 Hz), 6.23 (d, 1H, CH_arom._, *J* = 6.6 Hz), 3.76 (d, 2H, CH_2_, *J* = 11.4 Hz), 3.49–3.42 (m, 4H, CH_2_), 3.15–3.02 (m, 6H, CH_2_), 1.72–1.69 (m, 2H, CH_2_), 1.57–1.52 (m, 3H, CH_2_). ^13^C NMR (CDCl_3_) *δ* (ppm): 190.32, 165.58, 165.37, 149.52, 148.83, 141.58, 137.54, 135.13, 134.77, 134.39, 134.12, 133.94, 132.22, 130.47, 129.63 (2C), 129.41 (4C), 128.85 (2C), 128.49 (4C), 128.36 (2C), 127.24 (3C), 124.11, 123.53, 57.84, 57.65, 50.97, 50.86, 36.63, 34.50, 29.57, 26.48. ESI MS: *m*/*z* = 618.4 [M+H]^+^ (100 %).

### 4,10-Diphenyl-2-{4-[4-(pyridin-2-yl)piperazin-1-yl]butyl}-1*H*,2*H*,3*H*,5*H*-indeno[1,2-*f*]isoindole-1,3,5-trione (**13**)

Yield: 90 %, m.p. 219–220 °C. ^1^H NMR (DMSO-*d*_6_) *δ* (ppm): 8.14 (d, 1H, CH_arom._, *J* = 3.9 Hz), 7.82–7.74 (m, 1H, CH_arom._), 7.61 (t, 3H, CH_arom._, *J* = 3.6 Hz), 7.56–7.48 (m, 8H, CH_arom._), 7.40–7.31 (m, 2H, CH_arom._), 7.19–7.02 (m, 1H, CH_arom._), 6.84 (t, 1H, CH_arom._, *J* = 6.0 Hz), 6.23 (d, 1H, CH_arom._, *J* = 6.9 Hz), 4.37 (d, 2H, CH_2_, *J* = 15.0 Hz), 3.52–3.31 (m, 6H, CH_2_), 3.06–2.99 (m, 4H, CH_2_), 1.68–1.67 (m, 2H, CH_2_), 1.56–1.55 (m, 2H, CH_2_). ^13^C NMR (CDCl_3_) *δ* (ppm): 190.02, 165.63, 165.27, 153.84, 147.79, 141.44, 137.41, 135.58, 134.62, 134.29, 134.07, 133.68, 132.15, 130.32, 129.46 (2C), 129.39 (3C), 128.69 (2C), 128.38 (3C), 128.28, 128.20 (2C), 127.17 (3C), 124.46, 123.74, 52.35, 51.98, 48.79, 58.23, 36.96, 34.86, 27.62, 26.13. ESI MS: *m*/*z* = 619.4 [M+H]^+^ (100 %).

### 4,10-Diphenyl-2-[4-(4-{2-[2-(trifluoromethyl)phenyl]ethyl}piperazin-1-yl)butyl]-1*H*,2*H*,3*H*,5*H*-indeno[1,2-*f*]isoindole-1,3,5-trione (**14**)

Yield: 91 %, m.p. 232–233 °C. ^1^H NMR (DMSO-*d*_6_) *δ* (ppm): 7.46–7.65 (m, 2H, CH_arom._), 7.62–7.60 (m, 4H, CH_arom._), 7.56–7.44 (m, 9H, CH_arom._), 7.41–7.30 (m, 2H, CH_arom._), 6.23 (d, 1H, CH_arom._, *J* = 6.6 Hz), 3.46–3.37 (m, 4H, CH_2_), 3.34–3.09 (m, 14H, CH_2_), 1.66–1.65 (m, 2H, CH_2_), 1.57–1.55 (m, 2H, CH_2_). ^13^C NMR (CDCl_3_) *δ* (ppm): 190.18, 166.94, 166.57, 149.97, 148.22, 142.53, 137.16, 135.86, 134.51, 134.72, 134.58, 134.02 (2C), 132.48, 131.30, 129.64 (2C), 129.58 (3C), 129.48, 128.91 (2C), 128.75 (3C), 128.59, 128.27 (2C), 127.85 (2C), 127.03, 124.59, 123.61, 123.00, 59.89, 55.12, 55.01, 50.79, 50.83, 36.74, 34.95, 32.73, 29.48, 26.17. ESI MS: *m*/*z* = 714.3 [M+H]^+^ (100 %).

### 2-{4-[4-(2-Fluorophenyl)piperazin-1-yl]butyl}-4,10-diphenyl-1*H*,2*H*,3*H*,5*H*-indeno[1,2-*f*]isoindole-1,3,5-trione (**15**)

Yield: 88 %, m.p. 245–247 °C. ^1^H NMR (DMSO-*d*_6_) *δ* (ppm): 7.61 (t, 3H, CH_arom._, *J* = 3.9 Hz), 7.56–7.44 (m, 8H, CH_arom._), 7.41–7.30 (m, 2H, CH_arom._), 7.21–7.00 (m, 4H, CH_arom._), 6.23 (d, 1H, CH_arom._, *J* = 7.8 Hz), 3.50–3.37 (m, 8H, CH_2_), 3.21–3.08 (m, 4H, CH_2_), 1.70–1.68 (m, 2H, CH_2_), 1.58–1.53 (m, 2H, CH_2_). ^13^C NMR (CDCl_3_) *δ* (ppm): 191.47, 166.12, 165.97, 149.48, 148.57, 141.72, 137.16, 135.69, 134.38, 134.21, 134.09, 133.92, 132.46, 130.85 (2C), 129.36 (2C), 129.29 (3C), 128.63 (2C), 128.52 (3C), 128.47 (2C), 127.69 (4C), 124.82, 123.96, 57.06, 56.93, 50.46, 50.27, 36.12, 34.98, 29.58, 26.02. ESI MS: *m*/*z* = 636.4 [M+H]^+^ (100 %).

### 2-{4-[4-(4-Fluorophenyl)piperazin-1-yl]butyl}-4,10-diphenyl-1*H*,2*H*,3*H*,5*H*-indeno[1,2-*f*]isoindole-1,3,5-trione (**16**)

Yield: 93 %, m.p. 241–242 °C. ^1^H NMR (DMSO-*d*_6_) *δ* (ppm): 7.61 (t, 3H, CH_arom._, *J* = 3.9 Hz), 7.56–7.53 (m, 1H, CH_arom._), 7.51–7.48 (m, 3H, CH_arom._), 7.47–7.46 (m, 5H, CH_arom._), 7.41–7.30 (m, 2H, CH_arom._), 7.13–7.07 (m, 2H, CH_arom._), 7.03–6.98 (m, 2H, CH_arom._), 3.67 (d, 2H, CH_2_, *J* = 9.0 Hz), 3.47–3.42 (m, 4H, CH_2_), 3.06 (d, 6H, CH_2_, *J* = 8.4 Hz), 1.69–1.68 (m, 2H, CH_2_), 1.57–1.54 (m, 2H, CH_2_). ^13^C NMR (CDCl_3_) *δ* (ppm): 191.19, 166.58, 165.74, 149.53, 148.82, 141.13, 137.64, 135.97, 134.27, 134.09, 134.01, 133.84, 132.16, 130.76 (2C), 129.94 (3C), 129.59 (2C), 128.89 (3C), 128.72 (3C), 128.11 (2C), 127.75 (3C), 125.49, 123.52, 57.68, 57.51, 50.94, 50.00, 36.81, 34.86, 29.37, 26.97. ESI MS: *m*/*z* = 636.4 [M+H]^+^ (100 %).

### 2-{4-[4-(4-Chlorophenyl)piperazin-1-yl]butyl}-4,10-diphenyl-1*H*,2*H*,3*H*,5*H*-indeno[1,2-*f*]isoindole-1,3,5-trione (**17**)

Yield: 82 %, m.p. 248–249 °C. ^1^H NMR (DMSO-*d*_6_) *δ* (ppm): 7.61 (t, 3H, CH_arom._, *J* = 3.6 Hz), 7.56–7.53 (m, 1H, CH_arom._), 7.51–7.48 (m, 2H, CH_arom._), 7.47–7.45 (m, 5H, CH_arom._), 7.40–7.30 (m, 2H, CH_arom._), 7.31–7.27 (m, 2H, CH_arom._), 7.00 (d, 2H, CH_arom._, *J* = 9.0 Hz), 6.23 (d, 1H, CH_arom._, *J* = 7.5 Hz), 3.77 (d, 2H, CH_2_, *J* = 10.8 Hz), 3.49–3.72 (m, 4H, CH_2_), 3.07–3.01 (m, 6H, CH_2_), 1.68–1.66 (m, 2H, CH_2_), 1.57–1.52 (m, 2H, CH_2_). ^13^C NMR (CDCl_3_) *δ* (ppm): 190.64, 165.27, 165.11, 149.82, 148.56, 141.93, 137.14, 135.70, 134.31, 134.27, 134.03, 133.91, 132.27 (2C), 130.39 (2C), 129.79 (2C), 129.51 (3C), 128.88 (3C), 128.68 (3C), 128.02 (2C), 127.57 (2C), 124.69, 123.24, 57.49, 57.33, 50.17, 50.06, 36.94, 34.42, 29.96, 26.76. ESI MS: *m*/*z* = 652.4 [M+H]^+^ (100 %).

### 2-{4-[4-(2-Chlorophenyl)piperazin-1-yl]butyl}-4,10-diphenyl-1*H*,2*H*,3*H*,5*H*-indeno[1,2-*f*]isoindole-1,3,5-trione (**18**)

Yield: 75 %, m.p. 235–237 °C. ^1^H NMR (DMSO-*d*_6_) *δ* (ppm): 7.60 (t, 3H, CH_arom._, *J* = 3.6 Hz), 7.56–7.55 (m, 1H, CH_arom._), 7.53–7.48 (m, 2H, CH_arom._), 7.47–7.44 (m, 6H, CH_arom._), 7.40–7.31 (m, 3H, CH_arom._), 7.20–7.08 (m, 2H, CH_arom._), 6.23 (d, 1H, CH_arom._, *J* = 7.8 Hz), 3.51–3.28 (m, 6H, CH_2_), 3.19–3.07 (m, 6H, CH_2_), 1.70–1.68 (m, 2H, CH_2_), 1.58–1.53 (m, 6H, CH_2_). ^13^C NMR (CDCl_3_) *δ* (ppm): 190.30, 165.71, 165.49, 149.83, 148.79, 141.26, 137.44, 135.86, 134.92, 134.77, 134.51, 133.34 (2C), 132.58 (2C), 130.93 (2C), 129.81 (2C), 129.79 (2C), 128.73 (3C), 128.52 (3C), 128.39 (2C), 127.04 (2C), 124.82, 123.17, 58.14, 58.07, 52.58, 52.47, 35.97, 34.06, 29.74, 26.11. ESI MS: *m*/*z* = 652.4 [M+H]^+^ (100 %).

### Synthesis of 2-{4-[4-(2-metoxyphenyl)piperazin-1-yl]butyl}-4,10-diphenyl-1*H*,2*H*,3*H*,5*H*-indeno[1,2-*f*]isoindole-1,3,5-trione (**19**)

Yield: 79 %, m.p. 245–246 °C. ^1^H NMR (DMSO-*d*_6_) *δ* (ppm): 7.61 (t, 3H, CH_arom._, *J* = 3.6 Hz), 7.56–7.44 (m, 8H, CH_arom._), 7.41–7.31 (m, 2H, CH_arom._), 7.05–6.87 (m, 4H, CH_arom._), 6.23 (d, 1H, CH_arom._, *J* = 6.9 Hz), 3.79 (s, 3H, OCH_3_), 3.47–3.44 (m, 6H, CH_2_), 3.07–2.97 (m, 6H, CH_2_), 1.69–1.67 (m, 2H, CH_2_), 1.59–1.52 (m, 2H, CH_2_). ^13^C NMR (CDCl_3_) *δ* (ppm): 192.35, 165.07, 164.79, 149.81, 148.96, 141.13, 137.77, 135.42, 134.37, 134.26, 134.08, 133.11 (2C), 132.66 (2C), 130.72 (3C), 129.86, 129.72 (2C), 128.91 (3C), 128.54 (2C), 128.21 (3C), 127.75 (2C), 124.11, 123.59, 62.00, 58.84, 58.71, 52.97, 52.84, 35.06, 34.26, 29.59, 26.91. ESI MS: *m*/*z* = 648.3 [M+H]^+^ (100 %).

### 3-{4-[4-(2-Metoxyphenyl)piperazin-1-yl]butyl}3-azatricyclo[7.3.1.0^5,13^]trideca-(12),5,7,9(13),10-pentaene-2,4-dione (**20**) was obtained according to method presented previously (Hackling *et al*., 2003)

Yield: 63 %, m.p. 279–282 °C. ^1^H NMR (DMSO-*d*_6_) *δ* (ppm): 8.59–8.48 (d, 2H, CH_arom._, *J* = 8.1 Hz), 8.11 (d, 2H, CH_arom._, *J* = 7.8 Hz), 7.64 (t, 2H, CH_arom._, *J* = 7.6 Hz), 7.08–6.76 (m, 4H, CH_arom._) 4.56–4.17 (m, 2H, CH_2_), 3.87 (s, 3H, OCH_3_), 3,41–2.98 (m, 5H, CH_2_), 2.93–2.32 (m, 5H, CH_2_), 2.04–1.42 (m, 4H, CH_2_). ^13^C NMR (CDCl_3_) *δ* (ppm): 165.72, 159.08, 158.97, 140.62, 134.22, 134.17, 134.09, 133.74, 132.25, 130.14, 129.64, 129.53, 128.47, 128.38, 128.09, 127.48, 124.02, 123.61, 61.13, 60.95, 57.53, 51.27, 51.13, 41.37, 41.29, 26.96, 26.87. ESI MS: *m*/*z* = 344.6 [M+H]^+^ (100 %).

## Biological assays

### Cell-based assays

Cell-based assays were performed at Dipartimento di Scienze e Tecnologie Biomediche, Università di Cagliari, Monserrato, Italy.

### Test compounds

Compounds were dissolved in DMSO at 100 mM and then diluted in culture medium.

### Cells and viruses

Cell line and viruses were purchased from the American Type Culture Collection (ATCC). The absence of mycoplasma contamination was checked periodically by the Hoechst staining method. Cell line supporting the multiplication of human immunodeficiency virus type-1 (HIV-1) was the CD4+ human T-cells containing an integrated HTLV-1 genome (MT-4).

### Cytotoxicity assays

Cytotoxicity assays were run in parallel with antiviral assays.

Exponentially growing MT-4 cells were seeded at an initial density of 1 × 10^5^ cells/ml in 96-well plates in RPMI-1640 medium, supplemented with 10 % fetal bovine serum (FBS), 100 units/ml penicillin G, and 100 μg/ml streptomycin. Cell cultures were then incubated at 37 °C in a humidified 5 % CO_2_ atmosphere in the absence or presence of serial dilutions of test compounds. Cell viability was determined after 96 h at 37 °C by the 3-(4,5-dimethylthiazol-2-yl)-2,5-diphenyl-tetrazolium bromide (MTT) method (Pauwels *et al*., 1988).

### Antiviral assays

Compound’s activity against HIV-1 was based on inhibition of virus-induced cytopathogenicity in MT-4 cell acutely infected with a multiplicity of infection (m.o.i.) of 0.01. In brief, 50 μl of RPMI containing 1 × 10^4^ MT-4 cells were added to each well of flat-bottom microtitre trays, containing 50 μl of RPMI with or without serial dilutions of test compounds. Then, 20 μl of a HIV-1 suspension containing 100 CCID_50_ was added. After a 4-day incubation at 37 °C, cell viability was determined by the MTT method (Pauwels *et al*., 1988).

### In vitro ligand binding assays

Ligand studies with native 5-HT_1A_ receptor were conducted according to the methods previously described (Lewgowd *et al*., 2011).

### X-ray structure determination

Suitable crystals were mounted for measurements. The X-ray measurements were performed at 100(2) K on a KUMA CCD k-axis diffractometer with graphite-monochromated Mo Kα radiation (0.71073 Å). The crystals were positioned at 62.25 mm from the KM4CCD camera. The data were corrected for Lorentz and polarization effects, additionally absorption corrections were applied. Data reduction and analysis were carried out with the Kuma Diffraction (Wrocław, Poland) programmes (Oxford Diffraction CrysAlis CCD and CrysAlis RED, 2001). The structures were solved by direct methods (Sheldrick, 1990) and refined by using SHELXL (Sheldrick, 1997) The refinement was based on *F* ^2^ for all reflections except for those with very negative *F* ^2^. The weighted *R* factor, wR, and all goodness-of-fit *S* values are based on *F* ^2^. The non-hydrogen atoms were refined anisotropically. The hydrogen atoms were located from a difference map and were refined isotropically. The atomic scattering factors were taken from the International Tables (Wilson, 1992). Crystallographic data for the structures have been deposited with the Cambridge Crystallographic Data Centre as supplementary publication no. CCDC 913714-913719. Copy of the data can be obtained on application to CCDC, 12 Union Road, Cambridge CB2 1EZ, UK (email: deposit@ccdc.cam.ac.uk). 

#### X-ray crystal data for **2**

C_37_H_28_BrNO_3_, monoclinic space group *P*2_1_/*c*: *a* = 15.7066(8), *b* = 7.9750(4), *c* = 23.0807(12) Å, *β* = 100.366(4); *V* = 2843.9(3) Å^3^, *Z* = 4, *D*_calcd_ = 1.435 g/cm^3^; *μ* = 1.485 mm^−1^; *F*(000) = 1264. A total of 21,137 reflections were integrated in the *θ*-range of 2.71°–25.0° of which 5,007 were unique, leaving an overall *R*-merge of 0.041. For solution and refinement, 5,007 were considered as unique after merging for Fourier. The final agreement factors were *R*1 = 0.028 for 3,431 reflections with *F* > 4*σ*(*F*); *R*1 = 0.0501 and w*R*2 = 0.0553 for all the 5,007 data; GOF = 0.864. The residual electron density in the final difference Fourier does not show any feature above 0.33 e Å^−3^ and below −0.32 e Å^−3^.

#### X-ray crystal data for **6**

C_47_H_40_ClN_3_O_3_, monoclinic space group *P*2_1_/*n*: *a* = 11.8478(9), *b* = 23.8155(18), *c* = 13.0659(10) Å, *β* = 101.732(6); *V* = 3609.7(5) Å^3^, *Z* = 4, *D*_calcd_ = 1.344 g/cm^3^; *μ* = 0.155 mm^−1^; *F*(000) = 1536. A total of 27,540 reflections were integrated in the *θ*-range of 2.72°–25.0° of which 6,356 were unique, leaving an overall *R*-merge of 0.0653. For solution and refinement, 6,348 were considered as unique after merging for Fourier. The final agreement factors were *R*1 = 0.0339 for 2,916 reflections with *F* > 4*σ*(*F*); *R*1 = 0.0935 and w*R*2 = 0.1195 for all the 6348 data; GOF = 0.854. The residual electron density in the final difference Fourier does not show any feature above 0.22 e Å^−3^ and below −0.22 e Å^−3^.

#### X-ray crystal data for **7**

C_47_H_40_FN_3_O_3_, monoclinic space group *P*2_1_/*n*: *a* = 11.8103(4), *b* = 23.4267(5), *c* = 13.2359(3) Å, *β* = 96.196(2); *V* = 3640.67(17) Å^3^, *Z* = 4, *D*_calcd_ = 1.302 g/cm^3^; *μ* = 0.085 mm^−1^; *F*(000) = 1504. A total of 27,438 reflections were integrated in the *θ*-range of 2.8°–25.0° of which 6,394 were unique, leaving an overall *R*-merge of 0.0104. For solution and refinement, 6,394 were considered as unique after merging for Fourier. The final agreement factors were *R*1 = 0.0323 for 5,658 reflections with *F* > 4*σ*(*F*); *R*1 = 0.0365 and w*R*2 = 0.1276 for all the 6,394 data; GOF = 1.144. The residual electron density in the final difference Fourier does not show any feature above 0.24 e Å^−3^ and below −0.2 e Å^−3^.

#### X-ray crystal data for **11**

C_31_H_22_BrNO_3_, monoclinic space group *P*2_1_: *a* = 9.3851(7), *b* = 23.3058(14), *c* = 11.4605(7) Å, *β* = 106.711(7); *V* = 2400.9(3) Å^3^, *Z* = 4, *D*_calcd_ = 1.484 g/cm^3^; *μ* = 1.747 mm^−1^; *F*(000) = 1,096. A total of 9,877 reflections were integrated in the *θ*-range of 2.86°–26.0° of which 6,914 were unique, leaving an overall *R*-merge of 0.0318. For solution and refinement, 4,835 were considered as unique after merging for Fourier. The final agreement factors were *R*1 = 0.0633 for 4,665 reflections with *F* > 4*σ*(*F*); *R*1 = 0.1047 and w*R*2 = 0.1518 for all the 6,914 data; GOF = 1.049. The residual electron density in the final difference Fourier does not show any feature above 1.05 e Å^−3^ and below −0.96 e Å^−3^.

#### X-ray crystal data for **19**

C_41_H_36_Cl_2_N_3_O_3_, triclinic space group *P*-1: *a* = 11.4607(3), *b* = 12.0127(3), *c* = 13.7081(4) Å, α = 97.455(2), *β* = 103.874(2), γ = 105.357(2); *V* = 1728.71(8) Å^3^, *Z* = 2, *D*_calcd_ = 1.337 g/cm^3^; *μ* = 0.234 mm^−1^; *F*(000) = 728. A total of 19,541 reflections were integrated in the *θ*-range of 3.01°–25.0° of which 6,084 were unique, leaving an overall *R*-merge of 0.0173. For solution and refinement, 6,084 were considered as unique after merging for Fourier. The final agreement factors were *R*1 = 0.0351 for 4,789 reflections with *F* > 4*σ*(*F*); *R*1 = 0.0471 and w*R*2 = 0.0956 for all the 6,084 data; GOF = 1.077. The residual electron density in the final difference Fourier does not show any feature above 0.29 e Å^−3^ and below −0.25 e Å^−3^.

#### X-ray crystal data for 20

C_27_H_30_ClN_3_O_3_, triclinic space group *P*-1: *a* = 7.66540(10), *b* = 10.3318(2), *c* = 16.0440(3) Å, α = 96.0230(10), *β* = 93.910(2), γ = 106.740(2); *V* = 1203.60(4) Å^3^, *Z* = 2, *D*_calcd_ = 1.324 g/cm^3^; *μ* = 0.193 mm^−1^; *F*(000) = 508. A total of 13,968 reflections were integrated in the *θ*-range of 2.94°–25.0° of which 4,235 were unique, leaving an overall *R*-merge of 0.0149. For solution and refinement, 4,235 were considered as unique after merging for Fourier. The final agreement factors were *R*1 = 0.0267 for 3,532 reflections with *F* > 4*σ*(*F*); *R*1 = 0.0327 and w*R*2 = 0.0758 for all the 4,235 data; GOF = 1.068. The residual electron density in the final difference Fourier does not show any feature above 0.27 e Å^−3^ and below −0.21 e Å^−3^.

## Results and discussion

### Chemistry

#### Synthesis of *N*-butylarylpiperazinyl derivatives

**Scheme 1:**
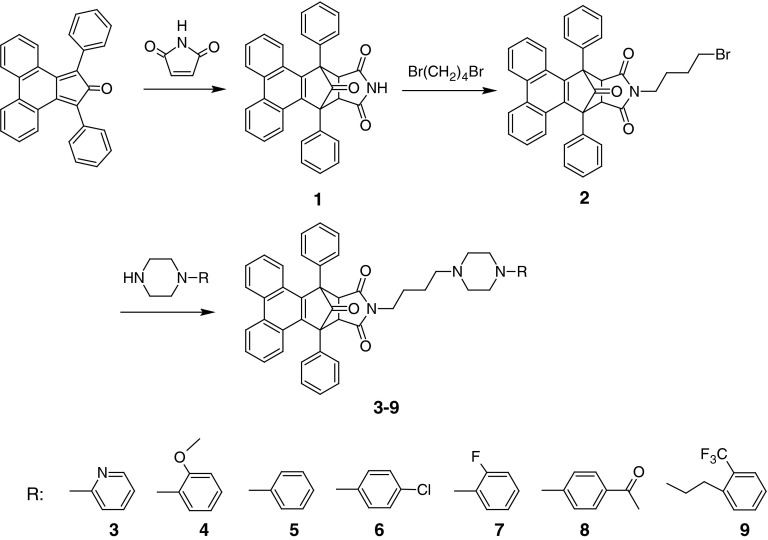
Synthesis of butylarylpiperazinyl derivatives of 1,16-diphenyl-19-azahexacyclo[14.5.1.0^2,15^.0^3,8^.0^9,14^.0^17,21^]docosa-2,3,5,7,8,9,11,13,14-nonaene-18,20,22-trione (**1**)

Two synthetic lines of N-substituted arylpiperazine derivatives were prepared. In the first path (Scheme 1), commercially available 1,3-diphenyl-2*H*-cyclopenta[*l*]phenanthren-2-one (“Phencyclone”) and maleimide were condensed in Diels–Alder reaction, and toluene was used as a solvent. After addition of 1,4-dibromobutane, 1,16-diphenyl-19-azahexacyclo[14.5.1.0^2,15^.0^3,8^.0^9,14^.0^17,21^]docosa-2,3,5,7,8,9,11,13,14-nonaene-18,20,22-trione was obtained **(1)**. Finally, synthesized 19-(4-bromobutyl)-1,16-diphenyl-19-azahexacyclo-[14.5.1.0^2,15^.0^3,8^.0^9,14^.0^17,21^]docosa-2,3,5,7,8,9,11,13,14-nonaene-18,20,22-trione **(2)** was used to obtain seven new complex arylpiperazines (**3**–**9**).

**Scheme 2:**
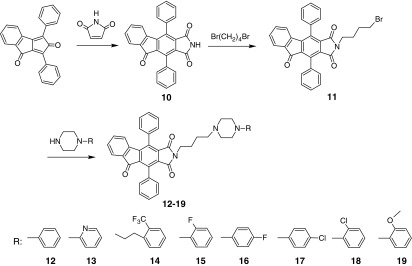
1,3-Diphenylcyclopenta[*a*]indene-2,8-dione as starting material for new synthetic route of complex arylpiperazines

In the second synthetic path (Scheme 2), “Indanocyclone” and maleimide were refluxed to give 4,10-diphenyl-1*H*,2*H*,3*H*,5*H*-indeno[1,2-*f*]isoindole-1,3,5-trione **(10)**. This step of synthesis shows different approaches (decarbonylation) of the condensation reaction between dienes and dienophiles.

The 2-(4-bromobutyl)-4,10-diphenyl-1*H*,2*H*,3*H*,5*H*-indeno[1,2-*f*]isoindole-1,3,5-trione **(11)** was obtained by condensation of 1,4-dibromobutane with above-mentioned complex imide in acetonitrile used as a solvent. The final step was to synthesize arylpiperazine derivatives by refluxing corresponding piperazines with 2-(4-bromobutyl)-4,10-diphenyl-1*H*,2*H*,3*H*,5*H*-indeno[1,2-*f*]isoindole-1,3,5-trione **(11).** Crude products (**12**–**19**) were purified and their hydrochlorides were made.

**Scheme 3:**
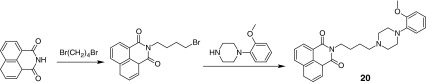
Synthesis of 3-{4-[4-(2-metoxyphenyl)piperazin-1-yl]butyl}3-azatricyclo[7.3.1.0^5,13^]trideca-(12),5,7,9(13),10-pentaene-2,4-dione (**20**)

In addition, the synthesis of 3-{4-[4-(2-metoxyphenyl)piperazin-1-yl]butyl}3-azatricyclo-[7.3.1.0^5,13^]trideca(12),5,7,9(13),10-pentaene-2,4-dione **(20)** was carried out. The compound was the subject of previous biological (5-HTR affinity) investigations (Scheme 3), however, the X-ray crystal analysis of the compound has not been published yet.

All obtained compounds were purified by flash chromatography. Elemental analysis, mass spectrometry, ^1^H NMR and ^13^C NMR spectra confirmed the identity of the products. For compounds **2** and **11**, also for hydrochlorides of **6**, **7**, **19**, and **20** X-ray analyses were done.

### Biology

#### Cytotoxicity and anti HIV-1 activity

Title compounds were tested in cell-based assay against the human immunodeficiency virus type-1 (HIV-1), using Efavirenz as reference inhibitor. The cytotoxicity was evaluated in parallel with the antiviral activity.

None of tested compounds showed selective antiviral activity against HIV-1. However compounds **10** and **14** turned out cytotoxic for exponentially growing MT4 cells in the low micromolar range (CC_50_ = 9 μM) (Table 1).

**Table 1:** Cytotoxicity and anti-HIV-1 activity of compounds **3**, **6**–**10**, and **12**–**19**

Compounds	MT-4	HIV-1_IIIB_
	CC_50_^a^	EC_50_^b^
**3**	90	>90
**6**	>100	>100
**7**	>100	>100
**8**	>100	>100
**9**	20	>20
**10**	9	>9
**12**	>100	>100
**13**	>100	>100
**14**	9	>9
**15**	>100	>100
**16**	>100	>100
**17**	>100	>100
**18**	>100	>100
**19**	>100	>100
Efavirenz	45	0.002

^a^Compound concentration (μM) required to reduce the viability of mock-infected MT-4 cells by 50 %, as determined by the MTT method

^b^Compound concentration (μM) required to achieve 50 % protection of MT-4 cells from the HIV-1-induced cytopathogenicity, as determined by the MTT method

### X-ray structural analyses

The crystal structures have been determined for three “phencyclone” derivatives **2**, **6**, and **7**. Their main skeleton resembles buspirone, but have more bulky maleimide fragment and in the case of **2** there is no piperazine moiety (*n*-butyl chain is terminated by bromine atom). In structures **6** and **7**, the aromatic fragment (*p*-chlorophenyl and *o*-fluorophenyl, respectively) is different from 2-pirymidinyl substituent in buspirone.

**Fig. 2:**
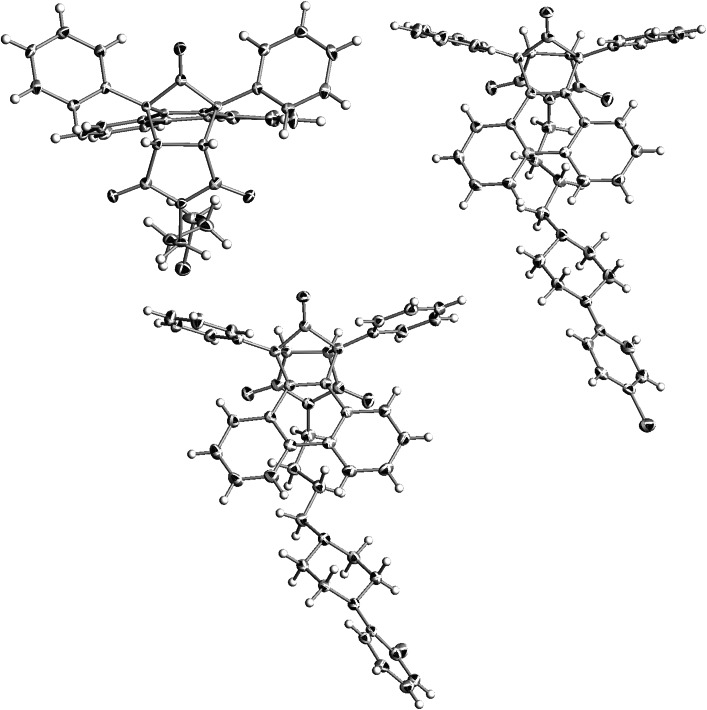
Crystal structures of **2**, **6**, and **7**. Thermal ellipsoids drawn at 50 % probability level

**Fig. 3:**
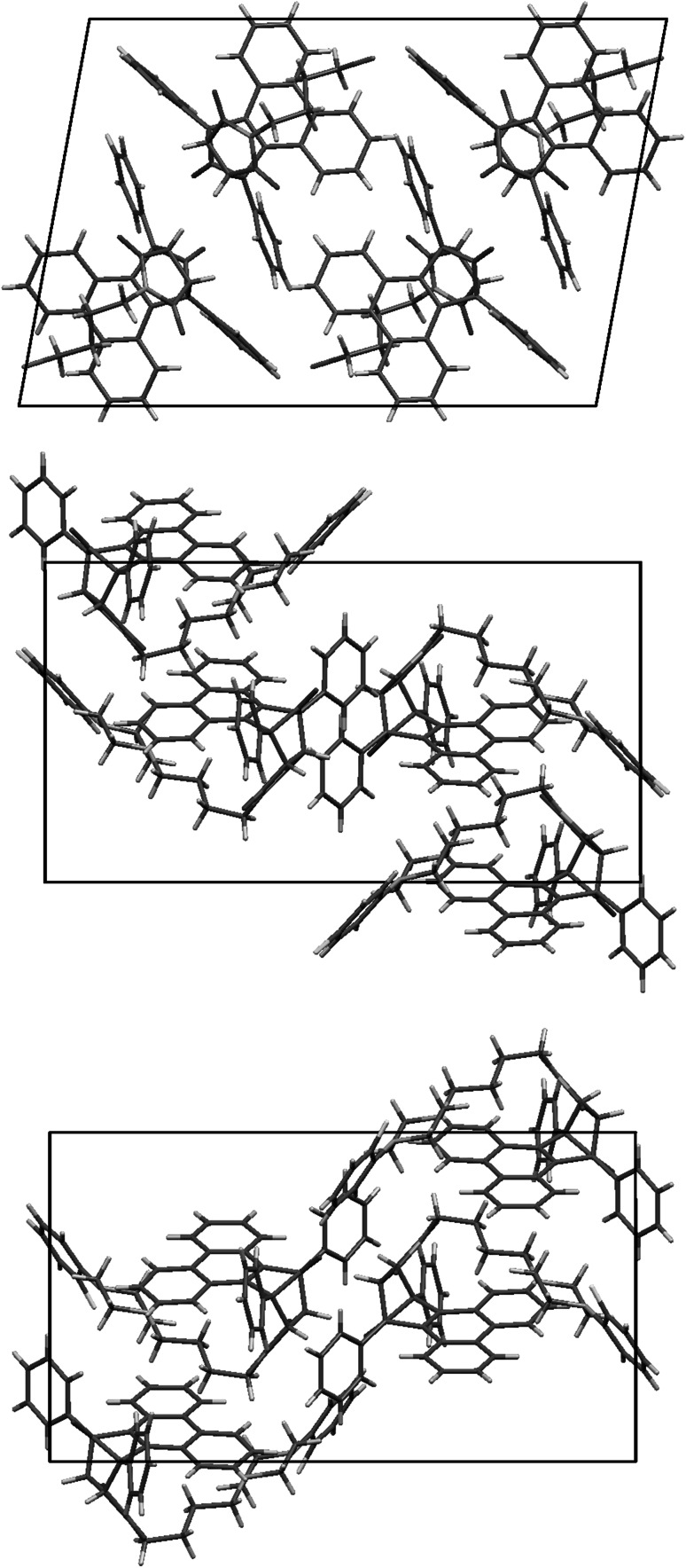
Crystal packing of **2**, **6**, and **7**

In all of these structures phenanthrene moiety forms a kind of “roof” over *n*-butyl chain, and phenyl rings are situated like “wings” directed outside (Fig. 2). In structures **6** and **7,** the piperazine moiety adopts chair conformation. All compounds crystallize in monoclinic system without solvent with one molecule in an asymmetric unit. Unit cell contains 4 molecules related by inversion center (Fig. 3).

**Fig. 4:**
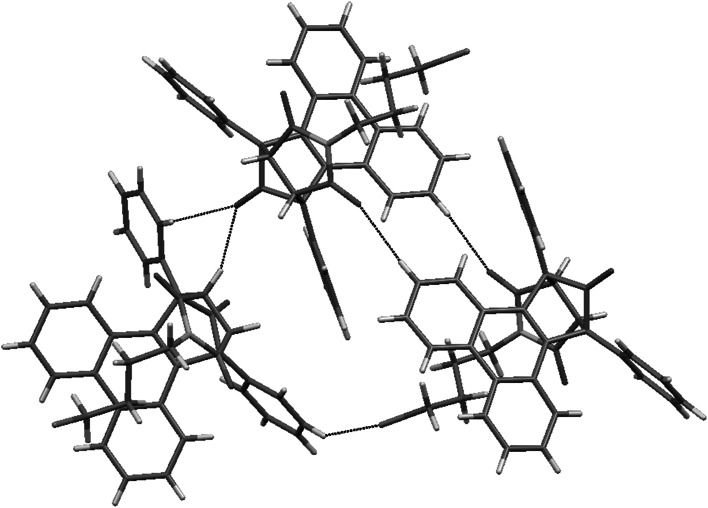
Short intermolecular contacts in crystal structure of **2**

**Fig. 5:**
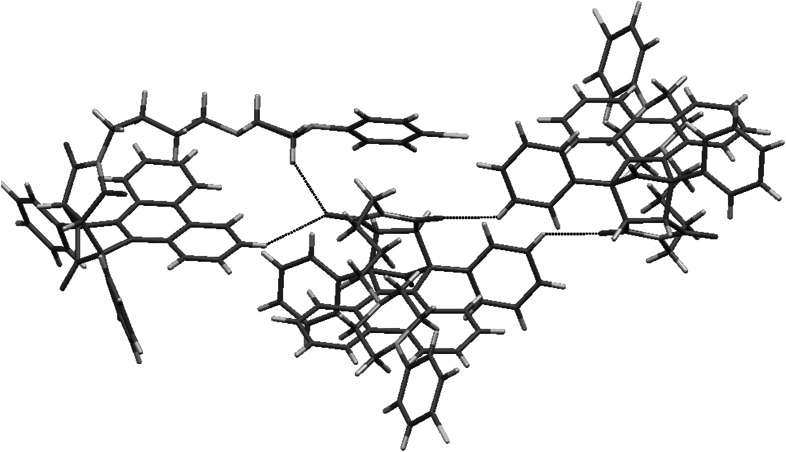
Short intermolecular contacts in crystal structure of **6**

**Fig. 6:**
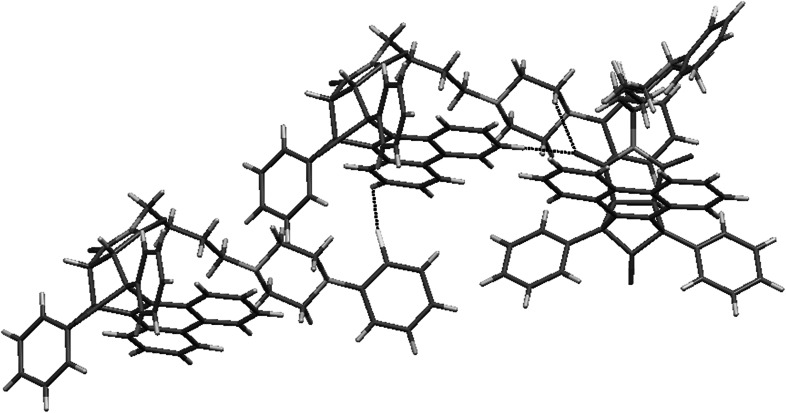
Short intermolecular contacts in crystal structure of **7**

The crystal structure of **2** is stabilized by two kinds of short interactions between C–H···O and C–H···Br (Fig. 4). In **6** there are three types of C–H···O contacts. The oxygen atom from maleimide moiety contacts with piperazine and phenanthrene fragments. Second one interacts with phenyl ring (Fig. 5). The structure of **7** shows similar C–H···O interactions and there is an additional short C–H···F contact (Fig. 6).

**Fig. 7:**
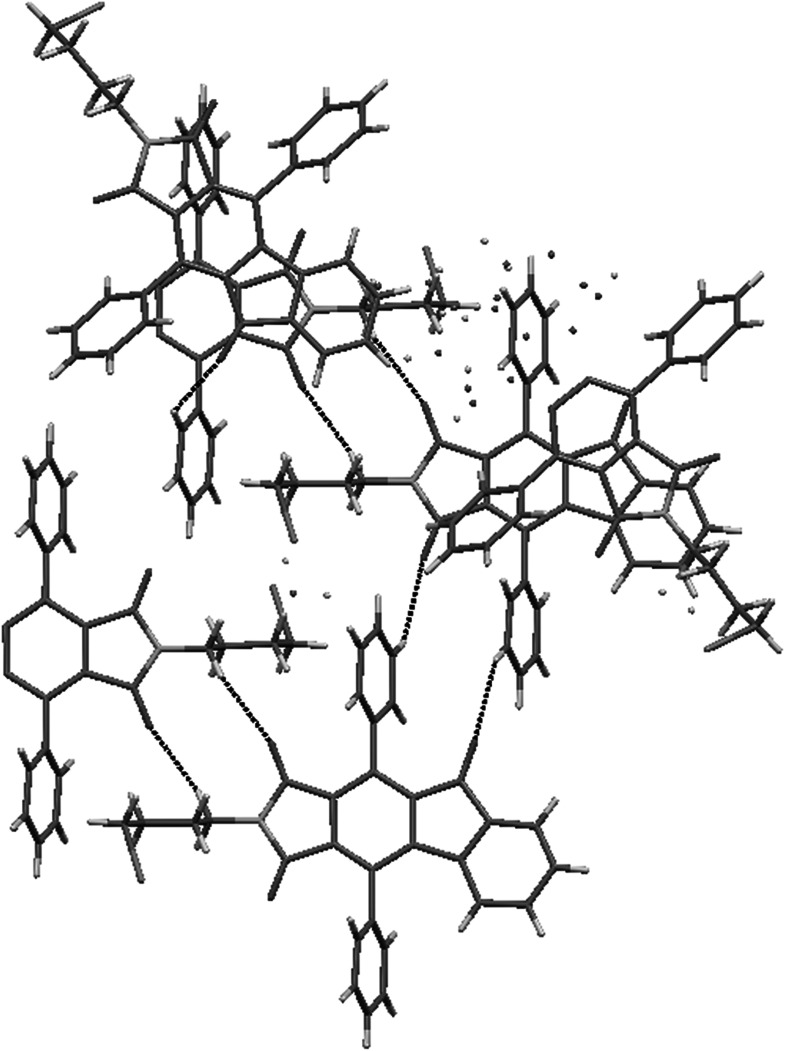
Crystal packing and short intermolecular contacts in crystal structure of **11**

Two crystal structures based on “Indanocyclone” **11** and **19** are disordered. Compound **11** crystallizes without solvent in monoclinic *P*2_1_ space group with two molecules in an asymmetric unit. The structure is a racemic twin in which one molecule is disordered. The disorder occurs in the *n*-butyl chain together with bromine atom and in the first phenyl ring of Indanocyclone. Two side phenyl rings are almost coplanar, the angle between mean best planes is 3.5°. There are three types of C–H···O interactions between maleimide oxygens and the *n*-butyl chain, as well as the side phenyl ring, and between oxygen from Indanocyclone moiety and the side phenyl ring (Fig. 7).

**Fig. 8:**
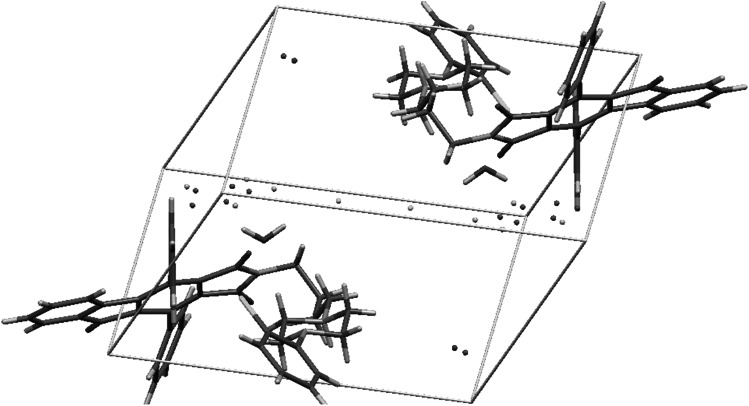
Crystal packing of **19**. Disordered phenyl ring showing π···π stacking with maleimide ring

Compound **19** crystallizes as a hydrochloride with one molecule of water in triclinic *P*-1 space group with one molecule in an asymmetric unit. Disorder occurs in first Indanocyclone phenyl ring and gives rise to π···π stacking between disordered benzene and maleimide rings. Two side phenyl rings are tilted with respect to each other by 24.8° (Fig. 8). The *n*-butyl chain adopts *cis* conformation with dihedral angle N1-C28-C29-C30 equal to 55.6.

**Fig. 9:**
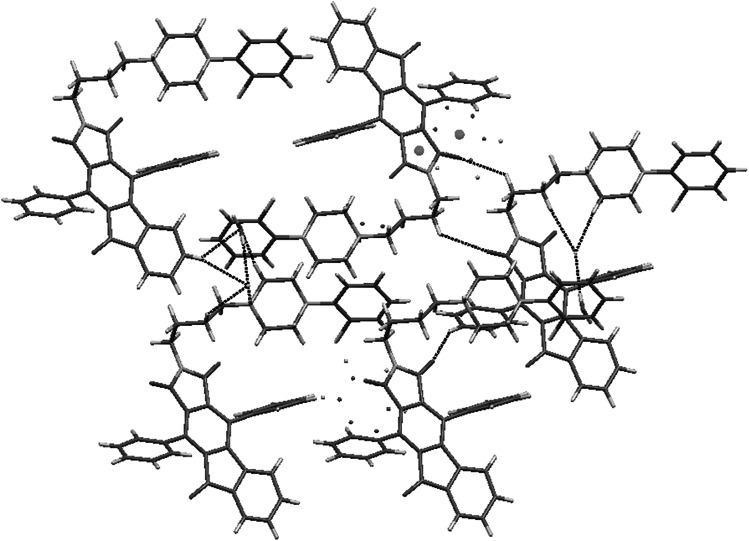
Short intermolecular contacts in crystal structure of **19**

The structure is stabilized by a set of N^+^H···Cl^−^ bonds between piperazine and chloride anions. There are two types of interactions between oxygens from maleimide moiety and C–H from butyl chain and Indanocyclone phenyl ring. Water molecule forms C–H···O bonds with piperazine and Indanocyclone phenyl ring. There are also O–H···Cl^−^ interactions (Fig. 9).

**Fig. 10:**
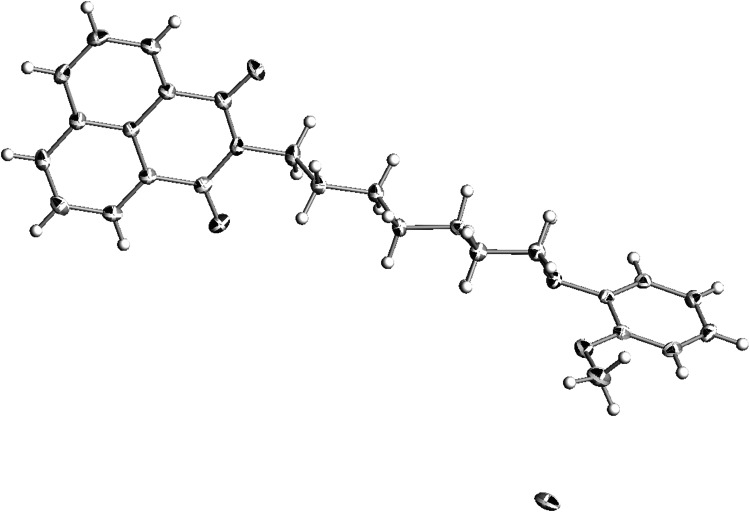
Crystal structure of **20**. Thermal ellipsoids drawn at 50 % probability level

**Fig. 11:**
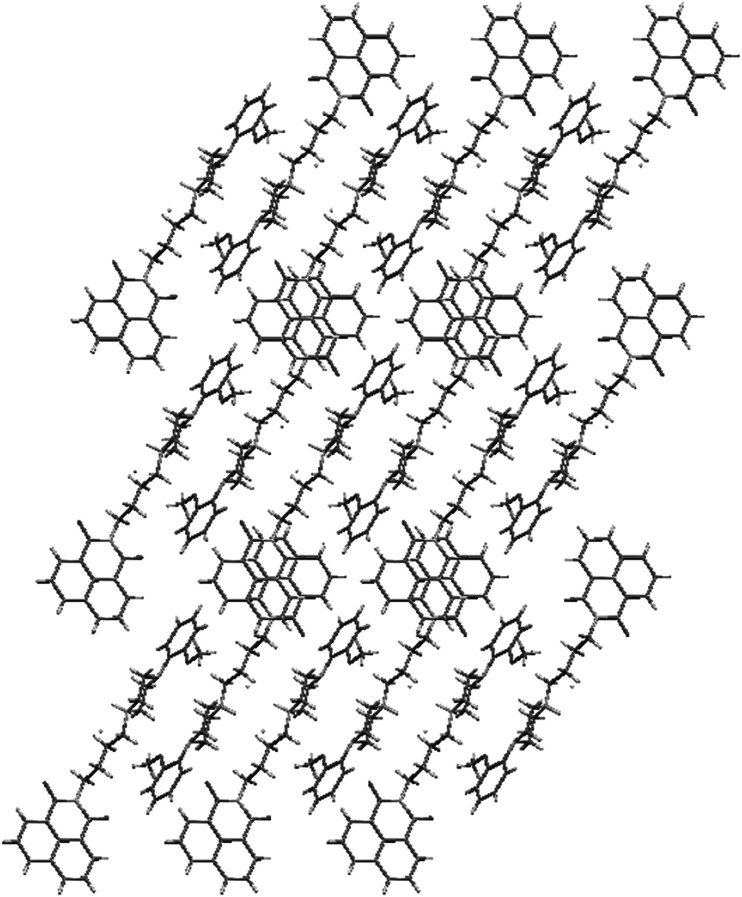
Crystal packing of **20**. View along *a* axis

**Fig. 12:**
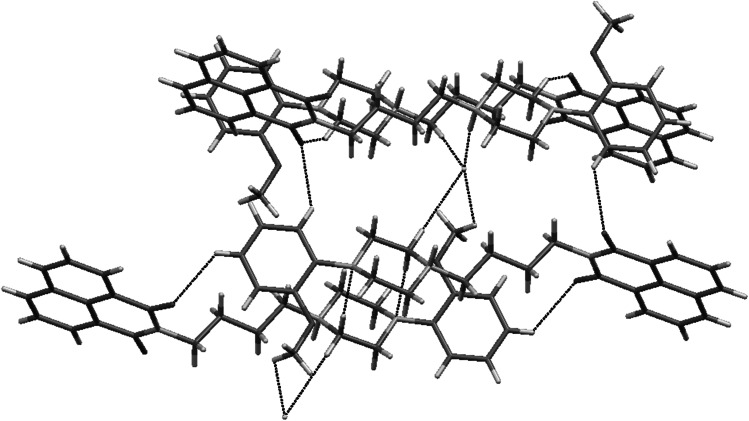
Short intermolecular contacts in crystal structure of **20**

Compound **20**, an analog of NAN-190, crystallizes in triclinic *P*-1 space group as a hydrochloride with one molecule in an asymmetric unit. The imide moiety is almost planar. The piperazine ring adopts chair conformation (Fig. 10). The crystal structure forms layers along *a* axis comprising of alternating molecules (Fig. 11). The structure is stabilized by N^+^H···Cl^−^ hydrogen bonds. In addition there are short contacts between chloride anion and C–H from the methoxy group, the butyl chain and the piperazine moiety. There are also interactions between oxygens from the imide fragment with C–H from piperazine and the methoxyphenyl ring (Fig. 12).

## Conclusions

Compounds **6**, **7**, **19**, and **20** fit well to the three-point pharmacophore model for 5-HT_1A_ receptor ligands (Chilmonczyk *et al.,* 1997). Apart from an aromatic ring and the basic nitrogen of piperazine, localized in the distance of 5.2–5.7 Å from a centroid, authors have found the third point essential for a ligand–receptor interaction—the carbonyl oxygen, expected in the distance of 7.07 Å from the center of an aromatic ring and 4.3 Å from N4 piperazine atom. Intramolecular distances measured for a set of 5-HT_1A_ receptor ligands by Chilmonczyk et al. were in the range of 7.93–12.37 Å (Centroid···O(1)), 3.95–7.16 Å (N(1)···O(1)), and 5.15–5.64 Å (Centroid···N(1)).

**Fig. 13:**
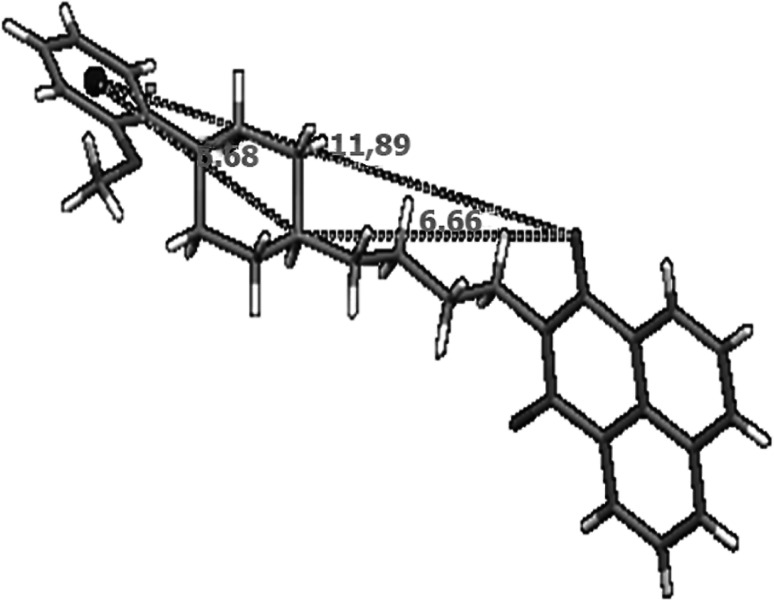
Molecular geometric parameters (in Å) observed in solid state for the derivative **20**

The values calculated for new arylpiperazine derivatives (**6**, **7**, **19**, and **20**) are in agreement with the presented three-point pharmacophore model (Table 2, Fig. 13). The distance between the center of the phenyl group and the imide oxygen (O1) is in the range of 8,13–11,89 Å. The measured distance of the protonated nitrogen (N1) and O1 atom is in the range of 4.06–6.66 Å. The value of centroid –N1 length is in a narrow range between 5.67 and 5.71 Å. Presented results suggest that compounds **6**, **7**, **19**, and **20** could serve as potential 5-HT_1A_ receptor ligands. They also prove that similar molecular values can be estimated for the derivative **4**. Although it is an exception from “the rule of five,” because of its high molecular weight, volume and log*P*, and low solubility log*S* (Table 3), the compound **4** possess moderate activity to the 5-HT_1A_ receptor.

**Table 2:** Selected intramolecular distances (Å) for arylpiperazine derivatives **6**, **7**, **19**, and **20**

	**6**	**7**	**19**	**20**
Centroid···O(1)	10.78	10.7	8.13	11.89
N(1)···O(1)	5.78	5.78	4.06	6.66
Centroid···N(1)	5.69	5.71	5.67	5.68

**Table 3:** Molecular descriptors calculated for representative 5-HT_1A_ receptor ligands and for selected synthesized derivatives (drug likeness prediction done via http://molsoft.com/mprop/)

Compound	Molecular weight (u)	Number of HBA	Number of HBD	log*P*	log*S* [log(moles/l]	PSA (Å^2^)	Volume (Å^3^)
Buspirone	385.25	5	0	2.09	−1.89	56.28	421.63
BMY-7378	385.24	4	0	3.14	−3.12	46.42	428.35
NAN-190	393.21	4	0	3.08	−4.16	44.93	415.76
4	725.33	5	0	6.82	−10.82	58.07	758.15
6	729.28	4	0	7.91	−11.22	49.46	769.80
7	713.31	4	0	7.33	−11.12	49.96	758.17
19	651.23	4	0	7.74	−10.79	49.75	646.73
20	443.22	4	0	4.25	−5.74	44.30	466.09

Structural data obtained for a set of long-chain arylpiperazine derivatives can serve for further investigations concerning ligands activity to metabotropic 5-HT receptors.

## References

[CR1] Ananda Kumar CS, Benaka Prasad SB, Vinaya K, Chandrappa S, Thimmegowda NR, Kumar YC, Swarup S, Rangappa KS (2009). Synthesis and in vitro antiproliferative activity of novel 1-benzhydrylpiperazine derivatives against human cancer cell lines. Eur J Med Chem.

[CR2] Bojarski AJ (2006). Pharmacophore models for metabotropic 5-HT receptor ligands. Curr Top Med Chem.

[CR3] Bronowska A, Leś A, Chilmonczyk Z, Filipek S, Edvardsen O, Ostensen R, Sylte I (2001). Molecular dynamics of buspirone analogues interacting with the 5-HT1A and 5-HT2A serotonin receptors. Bioorg Med Chem.

[CR4] Chilmonczyk Z, Szelejewska-Wozniakowska A, Cybulski J, Cybulski M, Koziol AE, Gdaniec M (1997). Conformational flexibility of serotonin1A receptor ligands from crystallographic data. Updated model of the receptor pharmacophore. Arch Pharm (Weinheim).

[CR5] Czopek A, Byrtus H, Kołaczkowski M, Pawłowski M, Dybała M, Nowak G, Tatarczyńska E, Wesołowska A, Chojnacka-Wójcik E (2010). Synthesis and pharmacological evaluation of new 5-(cyclo)alkyl-5-phenyl- and 5-spiroimidazolidine-2,4-dione derivatives. Novel 5-HT1A receptor agonist with potential antidepressant and anxiolytic activity. Eur J Med Chem.

[CR6] Filosa R, Peduto A, de Caprariis P, Saturnino C, Festa M, Petrella A, Pau A, Pinna GA, La Colla P, Busonera B, Loddo R (2007). Synthesis and antiproliferative properties of N3/8-disubstituted 3,8-diazabicyclo[3.2.1]octane analogues of 3,8-bis[2-(3,4,5-trimethoxyphenyl)pyridin-4-yl]methyl-piperazine. Eur J Med Chem.

[CR7] González-Gómez JC, Santana L, Uriarte E, Brea J, Villazón M, Loza MI, De Luca M, Rivas ME, Montenegro GY, Fontenla JA (2003). New arylpiperazine derivatives with high affinity for alpha1A, D2 and 5-HT2A receptors. Bioorg Med Chem Lett.

[CR8] Hackling A, Ghosh R, Perachon S, Mann A, Höltje HD, Wermuth CG, Schwartz JC, Sippl W, Sokoloff P, Stark H (2003). *N*-(omega-(4-(2-methoxyphenyl)piperazin-1-yl)alkyl)carboxamides as dopamine D2 and D3 receptor ligands. J Med Chem.

[CR9] Kerns EH, Di L (2008). Drug-like properties: concepts structure design and methods: from ADME to toxicity optimization.

[CR10] Kim MK, Lee HS, Kim S, Cho SY, Roth BL, Chong Y, Choo H (2012). 4-Aminoethylpiperazinyl aryl ketones with 5-HT_1_A/5-HT_7_ selectivity. Bioorg Med Chem.

[CR11] Klabunde T, Evers A (2005). GPCR antitarget modeling: pharmacophore models for biogenic amine binding GPCRs to avoid GPCR-mediated side effects. ChemBioChem.

[CR12] Leopoldo M (2004). Serotonin(7) receptors (5-HT(7)Rs) and their ligands. Curr Med Chem.

[CR13] Lepailleur A, Bureau R, Paillet-Loilier M, Fabis F, Saettel N, Lemaître S, Dauphin F, Lesnard A, Lancelot JC, Rault S (2005). Molecular modeling studies focused on 5-HT7 versus 5-HT1A selectivity. Discovery of novel phenylpyrrole derivatives with high affinity for 5-HT7 receptors. J Chem Inf Model.

[CR14] Lewgowd W, Bojarski AJ, Szczesio M, Olczak A, Glowka ML, Mordalski S, Stanczak A (2011). Synthesis and structural investigation of some pyrimido[5,4-*c*]quinolin-4(3*H*)-one derivatives with a long-chain arylpiperazine moiety as potent 5-HT(1A/2A) and 5-HT(7) receptor ligands. Eur J Med Chem.

[CR15] Lipinski CA, Lombardo F, Dominy BW, Feeney PJ (1997). Experimental and computational approaches to estimate solubility and permeability in drug discovery and development settings. Adv Drug Deliv Rev.

[CR16] López-Rodríguez ML, Porras E, Morcillo MJ, Benhamú B, Soto LJ, Lavandera JL, Ramos JA, Olivella M, Campillo M, Pardo L (2003). Optimization of the pharmacophore model for 5-HT7R antagonism. Design and synthesis of new naphtholactam and naphthosultam derivatives. J Med Chem.

[CR17] Nowak M, Kołaczkowski M, Pawłowski M, Bojarski AJ (2006). Homology modeling of the serotonin 5-HT1A receptor using automated docking of bioactive compounds with defined geometry. J Med Chem.

[CR18] Oprea TI (2002). Virtual screening in lead discovery: a viewpoint. Molecules.

[CR19] Oxford Diffraction Poland (2001). Oxford Diffraction CrysAlis CCD and CrysAlis RED.

[CR20] Pardo L, Deupi X, Dölker N, López-Rodríguez ML, Campillo M (2007). The role of internal water molecules in the structure and function of the rhodopsin family of G protein-coupled receptors. ChemBioChem.

[CR21] Pauwels R, Balzarini J, Baba M, Snoeck R, Schols D, Herdewijn P, Desmyter J, De Clercq E (1988). Rapid and automated tetrazolium-based colorimetric assay for the detection of anti-HIV compounds. J Virol Methods.

[CR22] Prandi A, Franchini S, Manasieva LI, Fossa P, Cichero E, Marucci G, Buccioni M, Cilia A, Pirona L, Brasili L (2012). Synthesis biological evaluation and docking studies of tetrahydrofuran- cyclopentanone- and cyclopentanol-based ligands acting at adrenergic α_1_- and serotonine 5-HT1A receptors. J Med Chem.

[CR23] Roth BL, Choudhary MS, Khan N, Uluer AZ (1997). High-affinity agonist binding is not sufficient for agonist efficacy at 5-hydroxytryptamine2A receptors: evidence in favor of a modified ternary complex model. J Pharmacol Exp Ther.

[CR24] Sheldrick GM (1990). Phase annealing in SHELX-90: direct methods for larger structures. Acta Cryst A.

[CR25] Sheldrick GM (1997). SHELXL97 program for the refinement of crystal structures.

[CR26] Siracusa MA, Salerno L, Modica MN, Pittalà V, Romeo G, Amato ME, Nowak M, Bojarski AJ, Mereghetti I, Cagnotto A, Mennini T (2008). Synthesis of new arylpiperazinylalkylthiobenzimidazole, benzothiazole, or benzoxazole derivatives as potent and selective 5-HT1A serotonin receptor ligands. J Med Chem.

[CR27] Sylte I, Bronowska A, Dahl SG (2001). Ligand induced conformational states of the 5-HT(1A) receptor. Eur J Pharmacol.

[CR28] Varin T, Gutiérrez-de-Terán H, Castro M, Brea J, Fabis F, Dauphin F, Aqvist J, Lepailleur A, Perez P, Burgueño J, Vela JM, Loza MI, Rodrigo J (2010). Phe369(738) at human 5-HT(7) receptors confers interspecies selectivity to antagonists and partial agonists. Br J Pharmacol.

[CR29] Vermeulen ES, Schmidt AW, Sprouse JS, Wikström HV, Grol CJ (2003). Characterization of the 5-HT(7) receptor. Determination of the pharmacophore for 5-HT(7) receptor agonism and CoMFA-based modeling of the agonist binding site. J Med Chem.

[CR30] Wilson AJC (1992). International tables for crystallography.

[CR31] Yang L, Xu X, Huang Y, Zhang B, Zeng C, He H, Wang C, Hu L (2010). Synthesis of polyhydroxylated aromatics having amidation of piperazine nitrogen as HIV-1 integrase inhibitor. Bioorg Med Chem Lett.

